# Cardiac-Restricted Expression of VCP/TER94 RNAi or Disease Alleles Perturbs *Drosophila* Heart Structure and Impairs Function

**DOI:** 10.3390/jcdd3020019

**Published:** 2016-05-24

**Authors:** Meera C. Viswanathan, Anna C. Blice-Baum, Tzu-Kang Sang, Anthony Cammarato

**Affiliations:** 1 Division of Cardiology, Department of Medicine, Johns Hopkins University, Ross 1050, 720 Rutland Avenue, Baltimore, MD 21205, USA; mcozhim1@jhmi.edu (M.C.V.); abliceb1@jhmi.edu (A.C.B.-B.); 2 Institute of Biotechnology & Department of Life Science, National Tsing Hua University, Hsinchu City 30013, Taiwan; tksang@life.nthu.edu.tw; 3 Department of Physiology, Johns Hopkins University, Ross 1050, 720 Rutland Avenue, Baltimore, MD 21205, USA

**Keywords:** TER94, cdc48, p97, myopathy, multisystem proteinopathy, IBMPFD

## Abstract

Valosin-containing protein (VCP) is a highly conserved mechanoenzyme that helps maintain protein homeostasis in all cells and serves specialized functions in distinct cell types. In skeletal muscle, it is critical for myofibrillogenesis and atrophy. However, little is known about VCP’s role(s) in the heart. Its functional diversity is determined by differential binding of distinct cofactors/adapters, which is likely disrupted during disease. VCP mutations cause multisystem proteinopathy (MSP), a pleiotropic degenerative disorder that involves inclusion body myopathy. MSP patients display progressive muscle weakness. They also exhibit cardiomyopathy and die from cardiac and respiratory failure, which are consistent with critical myocardial roles for the enzyme. Nonetheless, efficient models to interrogate VCP in cardiac muscle remain underdeveloped and poorly studied. Here, we investigated the significance of VCP and mutant VCP in the *Drosophila* heart. Cardiac-restricted RNAi-mediated knockdown of TER94, the *Drosophila* VCP homolog, severely perturbed myofibrillar organization and heart function in adult flies. Furthermore, expression of MSP disease-causing alleles engendered cardiomyopathy in adults and structural defects in embryonic hearts. *Drosophila* may therefore serve as a valuable model for examining role(s) of VCP in cardiogenesis and for identifying novel heart-specific VCP interactions, which when disrupted via mutation, contribute to or elicit cardiac pathology.

## 1. Introduction

Valosin-Containing Protein (VCP) is a type II AAA (ATPases associated with a variety of activities) enzymatic machine that converts energy from ATP hydrolysis into mechanical work [1,2]. VCP monomers assemble into a functioning macromolecular homo-hexameric complex in which six subunits form a ring around a central pore. It is highly conserved among eukaryotes, is ubiquitously expressed, and is extremely abundant. In animals, VCP accounts for more than 1% of total cellular protein. VCP is the most intensely investigated member of the AAA protein family, which can be attributed to its diverse biological roles. This diversity results from the enzyme’s promiscuity in substrate and cofactor binding [3,4]. It helps maintain proteostasis in all cells, in part, by governing critical steps during ubiquitin-proteasome system-mediated protein degradation and ER-associated degradation [5]. Other biological processes that involve VCP include membrane fusion, transcriptional activation, apoptosis, protein folding, and protein aggregation clearance [1,3,4,6]. VCP also performs specialized functions in distinct tissues. In skeletal muscle, for example, it is essential for myofibrillogenesis [3]. Critical roles during muscle atrophy and in the accelerated degradation of muscle proteins have also been recently identified [7]. The precise mechanisms underlying these roles, however, remain unknown. Similarly, a comprehensive understanding of how VCP mutations that cause human disease might alter tissue-specific functions and drive pathology is also lacking [8], which thereby necessitates the development and examination of an array of animal models.

Autosomal dominant mutations in VCP cause a debilitating form of inclusion body myopathy formerly known as inclusion body myopathy associated with Paget’s disease of the bone and fronto-temporal dementia (IBMPFD) [9], which more recently has been designated “multisystem proteinopathy” (MSP) [10]. MSP is an inherited degenerative disorder characterized by highly heterogeneous and variably penetrant phenotypes [3,9,10,11,12,13,14,15]. To date, more than 30 VCP missense mutations have been shown to cause MSP [16,17]. Clinical features include muscle weakness in mid-life, which advances to disabling weakness. Affected skeletal muscle contains “rimmed vacuoles” as well as myonuclear and sarcoplasmic inclusion bodies [18,19]. Weakness progresses to involve respiratory and heart muscles [17]. Patients frequently develop cardiomyopathy and typically die from respiratory or cardiac failure in their 40s to 60s [13,14,15,17]. The presence of cardiomyopathy is indicative of vital cardiovascular roles for the enzyme. Despite a vast expenditure of investigative effort to decipher numerous and unique functions of VCP in various tissues and in multiple cellular contexts, our comprehension regarding the roles of this enzymatic machine in heart muscle remains limited [20].

*Drosophila melanogaster*, the fruit fly, has proven to be an effective model for investigating VCP functions and MSP disease alleles [21,22,23,24,25]. *Drosophila* contains a single VCP homolog, TER94 [26], which shares 83% protein sequence identity with human VCP [21]. Expression of MSP *TER94* alleles in flies disrupts skeletal muscle integrity and performance, leads to the formation of inclusion body-like structures reminiscent of the rimmed vacuoles found in patients’ muscles, and causes progressive neurodegenerative defects [21]. Moreover, these models have helped uncover the nature of particular MSP-causing VCP mutations and a novel link between cellular ATP level and MSP pathogenesis and disease progression [21]. The fly is also well-suited for investigating cardiac-restricted roles of VCP. An array of genetic tools permits unprecedented temporal and spatial manipulation of the enzyme and its interacting partners. For example, the GAL4-UAS system permits targeted transgene expression [27]. Here, a transgene is located downstream of an “Upstream Activating Sequence” (UAS). It is inactive in the absence of the GAL4 transactivating protein. However, when flies carrying a UAS-transgene are mated with flies harboring a GAL4 transcriptional activator, the progeny inherit both genes and express the transgene in the same pattern as GAL4. Additionally, *Drosophila* has a short lifespan yet shares common mechanisms that determine aging rates and longevity [28,29]. This is especially advantageous for investigating phenotypes associated with progressive disorders, such as MSP, which typically require months to years to develop in higher organisms as opposed to weeks in *Drosophila*. The fly’s heart is composed of ~100 cardiomyocytes that form a simple tubular structure, and they share significant proteomic and ultrastructural homology with myocytes from vertebrate hearts [30]. Furthermore, since the fly does not rely on its heart for oxygenation, cardiac function can be severely compromised without necessarily inducing death. Thus, flies are highly tolerant of cardiac-specific genetic manipulation and serve as a valuable platform for *in vivo* analysis of myocardial defects that are lethal in other organisms.

To begin investigating cardiac-restricted roles of VCP we tested the hypotheses that TER94 is required for fly heart tube formation, maintenance, structure, and function and that mutations in conserved TER94 residues, when expressed only in the heart, initiate cardiac pathology and remodeling. RNAi-mediated *TER94* knockdown in adult hearts severely perturbed myofibrillar and cardiomyocyte organization and function. Conditional RNAi expression, post-development, induced extensive cardiac defects shortly after activation. Furthermore, expression of MSP disease-causing alleles triggered cardiomyopathy in adult flies and structural defects in embryonic hearts. These data support major roles for the enzyme during cardiogenesis and, in mature cells, in cardiomyocyte maintenance, which are likely disrupted by disease-causing mutations. Thus, *Drosophila* may serve as an efficient model for investigating VCP in myocardium, its tissue-specific interacting partners, and potential modifiers of the pathological response to disease-causing mutations.

## 2. Results

### 2.1. Cardiac-Specific Knockdown of TER94 Severely Affects Adult *Drosophila* Heart Structure and Function

**Figure 1:**
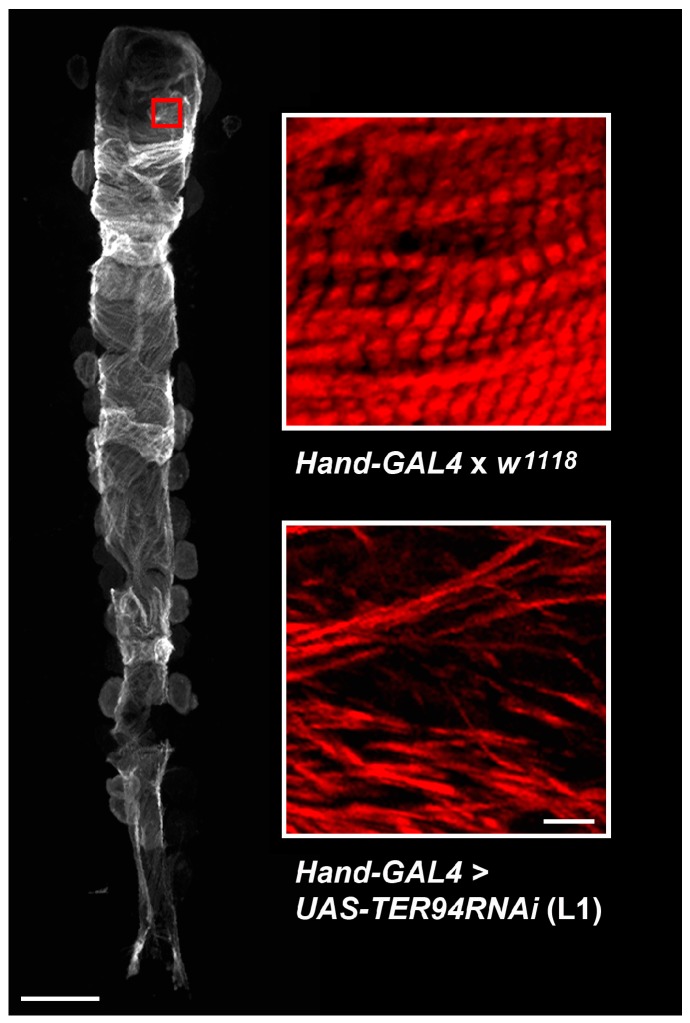
Heart-restricted *TER94^RNAi^* expression disrupts adult *Drosophila* cardiomyocyte and myofibrillar structure. (**Left**) Confocal micrograph of an adult *Drosophila melanogaster* cardiac tube with fluorescently-labeled F-actin. Scale bar = 100 μm. (**Right**) Myofibrils from a region (represented by the red box, left) of the anterior conical chamber of the heart. The myofibrils extended the length of cardiomyocytes of control *Hand^4.2^-GAL4 x w^1118^* flies at one week of age, and they exhibited highly organized, repetitive striations, which reflect well-ordered sarcomeres. In contrast, hearts of age-matched *Hand^4.2^-GAL4 > UAS-TER94^RNAi^* (L1) flies demonstrated an aberrant morphology overall. Their cardiomyocytes displayed severe myofibrillar disarray and completely lacked cross-striations, indicative of compromised sarcomerogenesis and/or disrupted myofibrillar maintenance and degeneration. This phenotype was consistently observed in all hearts of *Hand^4.2^-GAL4 > UAS-TER94^RNAi^* (L1) adult flies. Scale bar = 5 μm.

**Figure 2:**
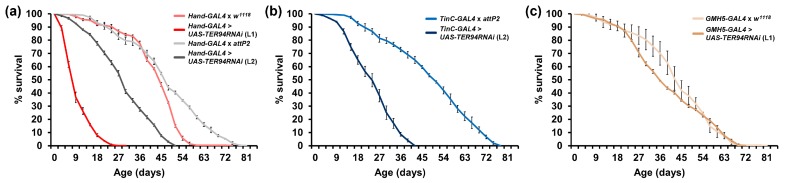
Heart-restricted *TER94^RNAi^* expression, driven by *Hand^4.2^-GAL4* or *TinC-GAL4*, reduces *Drosophila* lifespan. (**a**) Expression of *UAS-TER94^RNAi^* using the “strong” *Hand^4.2^-GAL4* driver shortened lifespan. *Hand^4.2^-GAL4* > *UAS-TER94^RNAi^* (L1) flies exhibited radically poor survivorship. *Hand^4.2^-GAL4* > *UAS-TER94^RNAi^* (L2) flies showed a better survival rate compared to (L1), albeit substantially reduced compared to the *Hand^4.2^-GAL4* x *attP2* control. (**b**) Expression of *UAS-TER94^RNAi^* (L2) using a second “strong” driver, *TinC-GAL4*, also resulted in severely shortened lifespan. *TinC-GAL4 > UAS-TER94^RNAi^* (L1) exhibited pupal lethality. (**c**) Expression of *UAS-TER94^RNAi^* (L1) using the “weak” *GMH5-GAL4* driver did not overtly alter lifespan. *n* = 300 flies/genotype.

**Figure 3:**
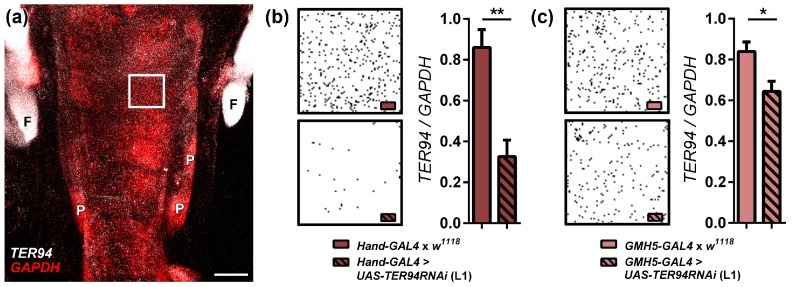
Heart-restricted *TER94^RNAi^* expression significantly reduces *TER94* mRNA. ViewRNA fluorescence *in situ* hybridization (ViewRNA FISH) was used to directly visualize and quantify *TER94* mRNA levels in the heart. (**a**) Conical chamber of a control *Drosophila* heart. White particles represent individual *TER94* transcripts and red particles represent individual *GAPDH* messages, which served as an endogenous control. Signals emanating from pericardial cells (P), fat (F), and associated skeletal muscles (not shown) were excluded from quantification. Scale bar = 25 μm. (**b**) Threshold adjusted *TER94* transcript signals from cardiomyocyte-restricted regions of the conical chamber (e.g., depicted by the white box in (**a**)) of *Hand^4.2^-GAL4 x w^1118^* and *Hand^4.2^-GAL4 > UAS-TER94^RNAi^* (L1) flies (**left**). *TER94* and *GAPDH* transcript levels were quantified using the ImageJ particle counter and *TER94*/*GAPDH* ratio determined (**right**). A large discernible reduction in *TER94*/*GAPDH* mRNA signals was evident in the *Hand^4.2^-GAL4 > UAS-TER94^RNAi^* (L1) hearts compared to *Hand^4.2^-GAL4 x w^1118^* hearts. Significance was assessed via unpaired *t*-test, ** *p* < 0.001, *n* = 7. (**c**) Threshold adjusted *TER94* transcript signals from conical chamber regions of *GMH5-GAL4 x w^1118^* and *GMH5-GAL4 > UAS-TER94^RNAi^* (L1) flies (**left**). A small but significant decrease in *TER94/GAPDH* ratio was identified in *GMH5-GAL4 > UAS-TER94^RNAi^* (L1) hearts compared to control (**right**). Significance was assessed via unpaired *t* test, * *p* < 0.05, *n* = 7.

**Figure 4:**
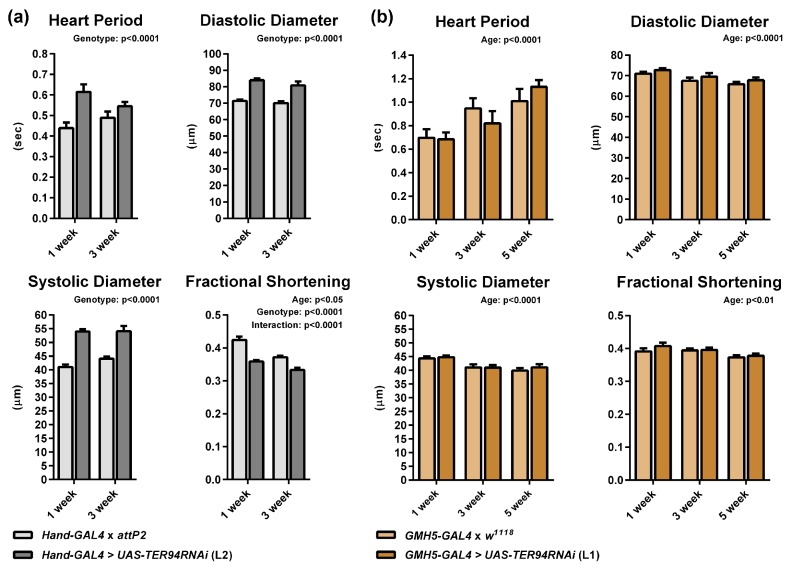
Excessive *TER94* knockdown causes a dilated cardiac phenotype. (**a**) Semi-automated optical heartbeat analysis of *Hand^4.2^-GAL4* > *UAS-TER94^RNAi^* (L2) flies at one and three weeks of age (1 Week: *n =* 25; 3 Week: *n =* 20) unveiled a dilated cardiac phenotype at both time points relative to control (1 Week: *n =* 23; 3 Week: *n =* 20). Diastolic and systolic diameters were significantly greater in *Hand^4.2^-GAL4* > *UAS-TER94^RNAi^* (L2) compared to *Hand^4.2^-GAL4* × *attP2*. In addition, age and *TER94* knockdown led to a significant reduction in fractional shortening. (**b**) Modest reduction of *TER94* in *GMH5-GAL4 > UAS-TER94^RNAi^* (L1) hearts did not prompt any significant alterations in cardiac performance in one-, three-, or five-week-old flies (1 Week: *n =* 21; 3 Week: *n =* 20; 5 Week: *n =* 20) compared to age-matched controls (1 Week: *n =* 24; 3 Week: *n =* 20; 5 Week: *n =* 22). Significance was assessed via two-way ANOVA, Bonferroni post-test.

Consistent with key myocardial roles for VCP, we previously identified high protein abundance in *Drosophila melanogaster* cardiac tubes [30], and our global *in vivo* RNAi screen distinguished TER94 as a possible regulator of heart performance [31]. To verify that TER94 is required for normal structure and function of the adult heart and its constituent cardiomyocytes, each of two *Drosophila* lines with UAS-controlled *TER94 RNAi* transgenes (*i.e.*, L1 and L2) was crossed with the cardiac-specific driver lines *Hand^4.2^-GAL4* or *TinC-GAL4*. The progeny expressed RNAi exclusively in myocardium. By one week of age *Hand^4.2^-GAL4 > UAS-TER94^RNAi^* (L1) adult heart tubes displayed aberrant cellular morphology (Figure 1). Specifically, in these flies, RNAi-mediated suppression of *TER94* expression disrupted the highly ordered packing of myofilaments into discrete striated contractile units, indicative of compromised sarcomerogenesis, and it rendered the hearts nonfunctional. *Hand^4.2^-GAL4 > UAS-TER94^RNAi^* (L1) accordingly exhibited a drastically shortened lifespan compared to control flies (Figure 2a). A relatively large reduction of *TER94* mRNA in adult cardiomyocytes was confirmed via fluorescence *in situ* hybridization (Figure 3a,b). *TinC-GAL4 > UAS-TER94^RNAi^* (L1) *Drosophila* were pupal lethal. To confirm these findings, a second *UAS-TER94^RNAi^* fly line (L2) was also crossed with the *Hand^4.2^-GAL4* and *TinC-GAL4* driver lines. The resulting progeny from both crosses exhibited markedly reduced lifespans relative to controls (Figure 2a,b). However, despite a similar reduction in *TER94* mRNA (Supplementary Figure S1), *Hand^4.2^-GAL4 > UAS-TER94^RNAi^* (L2) flies showed improved survival relative to *Hand^4.2^-GAL4 > UAS-TER94^RNAi^* (L1) flies (Figure 2a) and thus permitted extensive assessment of cardiac physiology at one and three weeks of age (Figure 4a). *Hand^4.2^-GAL4 > UAS-TER94^RNAi^* (L2) hearts exhibited a dilated phenotype at both time points compared to controls, specifically displaying significantly increased diastolic (1 Week: 84 ± 1 μm *vs.* 71 ± 4 μm; 3 Week: 81 ± 2 μm *vs.* 70 ± 1 μm) and systolic diameters (1 Week: 54 ± 1 μm *vs.* 41 ± 1 μm; 3 Week: 54 ± 2 μm *vs.* 44 ± 1 μm) and reduced fractional shortening (1 Week: 0.35 ± 0.01 *vs.* 0.42 ± 0.01; 3 Week: 0.33 ± 0.01 *vs.* 0.37 ± 0.01). While *TinC-GAL4 > UAS-TER94^RNAi^* (L2) flies survived to adulthood, their cardiac morphology and function were exceedingly poor and resembled that of *Hand^4.2^-GAL4 > UAS-TER94^RNAi^* (L1) hearts (data not shown). These data corroborate and extend our previous findings from a genome-wide RNAi screen that initially recognized cardiac function depends on normal expression of *TER94*, which when suppressed in the heart caused premature death [31].

### 2.2. Modest Cardiac-Specific Knockdown of TER94 Has No Effect on Lifespan or Cardiac Function

To determine if minor reductions of TER94 would produce a negative yet potentially mild effect on overall cardiac performance and survival, flies expressing the relatively weak *GMH5-GAL4* cardiac-specific driver were crossed with *UAS-TER94^RNAi^* (L1) *Drosophila*. *GMH5-GAL4 > UAS-TER94^RNAi^* (L1) cardiomyocytes were characterized by a slight but significant reduction of *TER94* mRNA (Figure 3c). No difference was detected between lifespans of *GMH5-GAL4 > UAS-TER94^RNAi^* (L1) flies and controls (Figure 2c). Furthermore, no discernable differences in cardiac performance indices between *GMH5-GAL4 > UAS-TER94^RNAi^* (L1) and controls were identified in one-, three-, and five-week-old flies (Figure 4b). Thus, mild expression of *UAS-TER94^RNAi^* (L1) in the *Drosophila* heart using the *GMH5-GAL4* driver may not silence a sufficient amount of mRNA to have significantly negative effects on myocardial structure or function.

### 2.3. Heart-Specific Knockdown of TER94 Post-Development Affects Cardiac Structure and Function

**Figure 5:**
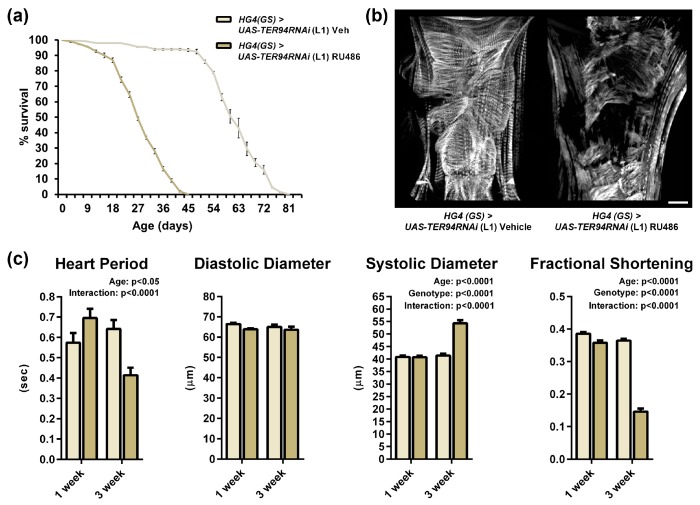
Conditional expression of *TER94^RNAi^* using the cardiac-specific RU486-inducible *Hand^4.2^-GS-GAL4* driver disrupts the adult *Drosophila* heart post-development. (**a**) Post-development cardiac-restricted knockdown of *TER94* (induced by 100 μg/mL RU486) significantly shortened lifespan compared to that of cohorts on vehicle. *n* = 300 flies/condition. (**b**) Confocal micrographs showing extensive myofibrillar degeneration with substantial loss of sarcomeric material in the conical chamber of a fly following *UAS-TER94^RNAi^* (L1) expression relative to control. Images were taken after flies were maintained for three weeks on food supplemented with 100 μg/mL RU486 to activate RNAi compared to siblings maintained on food with vehicle (100% ethanol). The aberrant myocardial phenotype was observed in all RU486 fed flies. Scale bar = 25 μm. (**c**) Cardiac performance of flies on drug and expressing *UAS-TER94^RNAi^* (L1) for one week post-eclosion (*n* = 21) was unaltered compared to flies on vehicle (*n* = 20). In contrast, after three weeks on drug, flies with *TER94* knockdown (*n* = 20) displayed poorly functioning hearts with significantly large systolic diameters and reduced fractional shortening *vs.* control (*n* = 30). Significance was assessed via two-way ANOVA, Bonferroni post-test.

To determine if the negative effects observed in adult *Hand^4.2^-GAL4* *>* or *TinC-GAL4 > UAS-TER94^RNAi^* flies result exclusively from impaired heart development, and to discern if the enzyme is required for mature cardiomyocyte maintenance, an RU486-inducible cardiac-restricted driver, *Hand^4.2^-GeneSwitch-GAL4* (*Hand^4.2^-GS-GAL4*), was crossed with *UAS-TER94^RNAi^* (L1). At two days post-eclosion, adult progeny were placed on food supplemented with 100 μg/mL RU486 (dissolved in 100% ethanol) to activate the RNAi transgene or vehicle (100% ethanol). Lifespan was abruptly shortened in flies aged on the drug compared to sibling controls aged on vehicle (Figure 5a). After one week, however, no morphological differences or reductions in cardiac performance in flies on drug relative to those on vehicle were observed. After three weeks of aging on drug, flies showed extensive cardiac remodeling similar to that observed in *Hand^4.2^-GAL4 > UAS-TER94^RNAi^* (L1) hearts at one week (Figure 5b and Figure 1). Most cardiomyocytes of the conical chamber showed either a complete loss of sarcomeric material or disorganized, poorly-striated myofibrils. Cardiac performance declined accordingly compared to those on vehicle, as manifested by significantly reduced fractional shortening (0.15 ± 0.01 *vs.* 0.36 ± 0.01) governed by significantly larger systolic diameters (54 ± 1 μm *vs.* 41 ± 1 μm) (Figure 5c). Importantly, wildtype adult flies similarly treated with 100 μg/mL of RU486 for three weeks exhibited no difference in cardiac diameters or fractional shortening relative to flies exposed to vehicle (Supplementary Figure S2). These data are consistent with those obtained by Tricoire *et al.* (2013) [32]. Thus, excessive reduction of cardiac *TER94* via RNAi knockdown, both continuously and conditionally, resulted in structural defects, poor heart function, and decreased lifespan. In sum, the above findings support a vital requirement for relatively high VCP expression for both cardiogenesis and cardiomyocyte maintenance throughout life. Naturally occurring mutations in VCP, which impair the enzyme’s functions, may likewise deleteriously impact heart performance and trigger cardiac pathology.

### 2.4. Expression of TER94 MSP Alleles Engenders a Restrictive Cardiac Phenotype in Adult Flies

**Figure 6:**
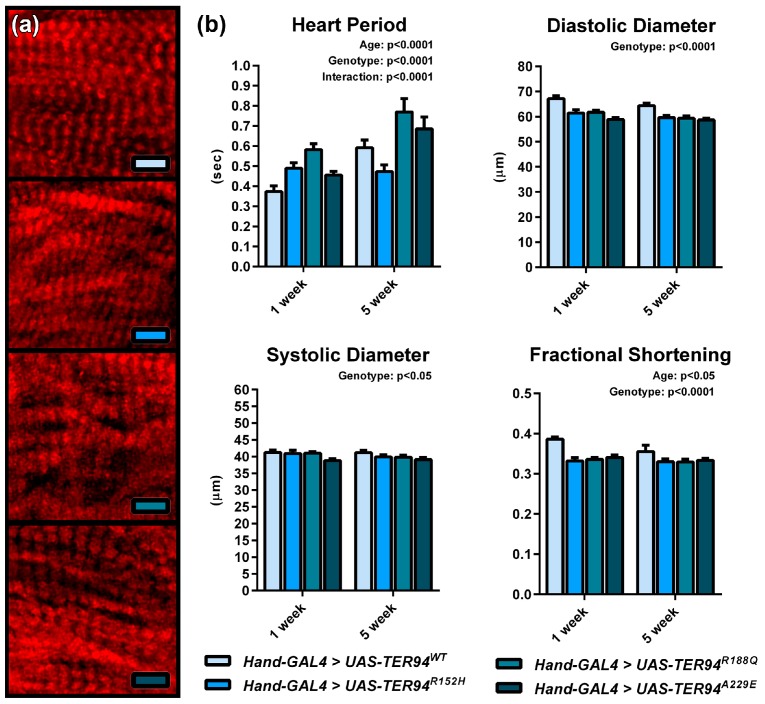
Expression of *TER94* MSP disease alleles in *Drosophila* causes cardiomyopathy. (**a**) Confocal micrographs of myofibrils from the conical chamber of the hearts of five-week-old *Hand^4.2^-GAL4* > *UAS-TER94^WT^*, *Hand^4.2^-GAL4* > *UAS-TER94^R152H^*, *Hand^4.2^-GAL4* > *UAS-TER94^R188Q^*, and *Hand^4.2^-GAL4* > *UAS-TER94^A229E^* flies. Compared to wildtype *TER94*, expression of the MSP *TER94* alleles did not noticeably alter cardiomyocyte myofibrillar organization. Scale bars’ color demarcates genotype (see (**b**)) and = 5 μm. (**b**) Assessment of cardiac function in flies expressing MSP *TER94* alleles revealed restrictive cardiac physiology as early as one week of age. All three mutant transgenic lines displayed significantly restricted diastolic diameters resulting in reduced fractional shortening. The phenotype did not worsen drastically with age but all mutant hearts remained significantly restricted relative to age-matched controls. Significance was assessed via two-way ANOVA, Bonferroni post-test, *n* = 40–42 flies/genotype or age group.

Although a prevalent phenotype associated with MSP is skeletal muscle myopathy, patients exhibit an increased potential for cardiomyopathy and heart failure [13,14,15,17]. Three *TER94* transgenic *Drosophila* lines were generated to model discrete human VCP mutations associated with MSP [21]. They recapitulate many hallmarks of the human disease. To determine if these specific TER94 mutations affect myofibrillar structure in cardiomyocytes and/or lead to cardiomyopathy, *Drosophila* containing a UAS-controlled wildtype *TER94* (*UAS-TER94^WT^*) transgene, or a transgene with the MSP-causing mutations TER94 R152H (*UAS-TER94^R152H^*), R188Q (*UAS-TER94^R188Q^*), or A229E (*UAS-TER94^A229E^*) were crossed with flies harboring the *Hand^4.2^-GAL4* driver. Unlike what was observed following RNAi expression, expression of wildtype *TER94* or MSP *TER94* alleles seemingly had no effect on sarcomeric development or organization in the progeny (Figure 6a). All three mutant transgenic lines exhibited myofibrillar striations that were indistinguishable from those of control. At one week, however, hearts expressing the *UAS-TER94^R152H^*, *UAS-TER94^R188Q^*, or *UAS-TER94^A229E^* mutant alleles displayed significantly restricted diastolic diameters compared to control hearts (1 Week: 61 ± 1 μm, 62 ± 1 μm, and 59 ± 1 μm, respectively, *vs.* 67 ± 1 μm) (Figure 6b). By five weeks, the restriction did not worsen drastically in the hearts of all three mutants compared to one week but remained significantly restricted *vs.* age-matched control hearts (5 Week: 60 ± 1 μm, 59 ± 1 μm, and 59 ± 1 μm, respectively, *vs.* 64 ± 1 μm) (Figure 6b). Because of mutant diastolic restriction, fractional shortening was also significantly reduced relative to controls (1 Week: 0.33 ± 0.01, 0.34 ± 0.01, and 0.34 ± 0.01, respectively, *vs.* 0.39 ± 0.01; 5 Week: 0.33 ± 0.01, 0.33 ± 0.01, and 0.33 ± 0.01, respectively, *vs.* 0.36 ± 0.01) (Figure 6b). These results demonstrate that heart-specific expression of mutant isoforms of TER94, which are analogous to MSP-causing variants in humans, induces cardiac remodeling and myopathic responses.

### 2.5. Expression of TER94 MSP Alleles in Muscle Progenitor Cells Causes Cardiac Defects in the Embryonic Heart

**Figure 7:**
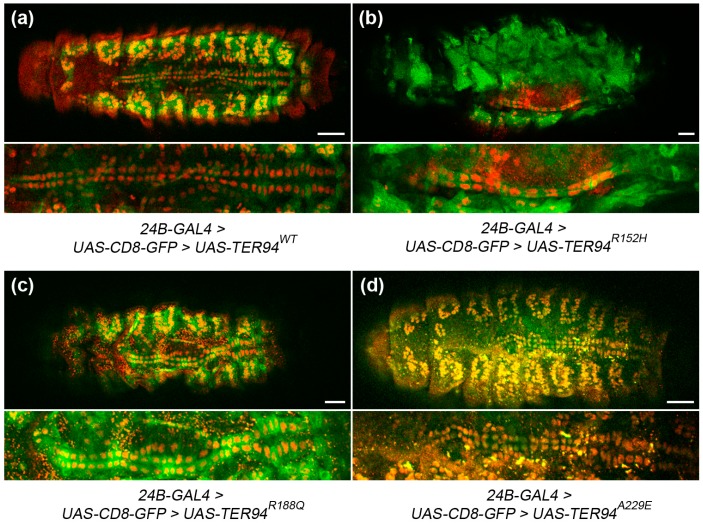
Overexpressing *TER94* MSP mutant alleles in muscle progenitor cells causes structural defects in the *Drosophila* embryonic heart. Dorsal views of stage 16 embryos expressing membrane-tethered CD8-GFP and the indicated *TER94* allele using the *24B-GAL4* driver. Embryos were stained with anti-dMef2 antibody (**red**) to label the dorsal vessel (enlarged in lower panels) and somatic muscle cells. (**a**) In embryos that expressed *UAS-TER94^WT^*, the two rows of cardioblasts aligned contralaterally to meet at the dorsal midline forming the dorsal vessel, and somatic cells were organized in a regular pattern. (**b**–**d**) In contrast, expression of the MSP-inducing mutant *TER94* alleles *UAS-TER94^R152H^*, *UAS-TER94^R188Q^*, or *UAS-TER94^A229E^* resulted in a disordered alignment of the cardiac cells and an abnormally twisted conformation of the dorsal vessel. Moreover, disorganization of the somatic muscle cell pattern at the body segments was evident. The mutant phenotypes appeared modestly variable in severity and were highly penetrant. Nearly all embryos examined displayed aberrant somatic and/or cardiac muscular patterning. Scale bars = 25 μm.

To determine if MSP-inducing alleles have an effect on early *Drosophila* cardiac development, UAS-controlled wildtype and mutant *TER94* transgenes were expressed via the myoblast-specific driver, 24B-GAL4, which inherently co-expressed CD8-GFP. Stage 16 live embryos expressing *UAS-TER94^WT^*, *UAS-TER94^R152H^*, *UAS-TER94^R188Q^*, or *UAS-TER94^A229E^* were collected and stained with anti-dMef2 antibody to help visualize potential structural defects in the dorsal heart and the somatic musculature. *24B-GAL4 > UAS-CD8-GFP > UAS-TER94^WT^* *Drosophila* exhibited an ordered, two-row contralateral arrangement of cardioblasts and a regular segmental arrangement of somatic cells (Figure 7a). In contrast, embryos expressing MSP-causing mutant *TER94* showed disorganized pairing of myocardial cells with an aberrant twisting of the heart (Figure 7b–d). In addition, disrupted organization of the intersegmental somatic muscle cell pattern was evident. These results show that *24B-GAL4*-mediated expression of MSP mutant isoforms of TER94 induces cardiac remodeling and myopathic responses in the early developing embryonic *Drosophila* heart and somatic skeletal musculature.

## 3. Discussion

VCP is an abundant, multi-functional, and highly conserved enzyme. Very few studies have focused on the roles of this enzymatic machine in heart muscle. It thus remains crucial to understand how VCP influences normal myocardial physiology. Moreover, comprehending how disease-associated mutations in the protein disrupt cardiac morphology and performance, which contribute to heart failure and early death, is also imperative.

VCP-associated MSP is an inherited, heterogeneous, and progressive syndrome characterized by myopathy, dementia, motor neuron disease, and/or Paget disease of bone [3,9,10,11,12,13,14,15]. Predominantly, afflicted patients present gradual skeletal muscle weakness and die from heart and respiratory failure. The potential for cardiomyopathy and heart failure underscores critical roles for VCP in cardiac muscle, which are likely perturbed during disease [13,14,15]. As with other myopathies, in order to help take additional steps toward management and treatment of MSP, it is necessary to establish novel model systems suitable for high throughput and comprehensive investigation of the protein in the heart and in the pathogenesis of VCP-related cardiac anomalies.

Here, we investigated requisite functions of VCP/TER94 in the *Drosophila* heart tube. Our results represent the first demonstration of the cardiac autonomous responses to VCP knockdown and MSP-associated mutations, *in vivo*. They suggest the fly heart may serve as a unique system to investigate MSP-associated heart failure precipitated by VCP mutations. Suppression of *TER94* expression using two independent UAS-RNAi lines in conjunction with the relatively strong cardiac-specific GAL4 driver lines, *Hand^4.2^-GAL4 or TinC-GAL4*, severely disturbed adult heart structure and function. In certain instances, e.g., pupal lethality, our data indicate *TER94* knockdown substantially impairs cardiac development. We additionally observed reduced lifespans, which is also consistent with severely compromised heart performance. *In situ* live-video imaging and assessment of myocardial physiology verified impaired function. These alterations can be attributed to, at least in part, poorly organized contractile elements. The loss of discrete actin-based striations along myofibrils throughout the cardiomyocytes suggests impaired sarcomerogenesis, and possibly maintenance of the highly ordered cytoskeletal structures. Relatively low constitutive expression of *TER94* RNAi, however, had no obvious effects on myocardial physiology, myofibrillar order, or lifespan. These findings imply that high cardiac *TER94* expression levels normally afford an important functional reserve such that mild loss of the enzyme can be tolerated without overt biological consequences. Conditional *TER94* knockdown, post-cardiac tube development, initially had minimal effects on the *Drosophila* heart. Major defects in cardiac function and myofibrillar organization, however, were identified within weeks of RNAi activation. Use of the RU486-inucuble driver ruled out the possibility that the hearts of *Hand^4.2^-GAL4 >* or *TinC-GAL4 > UAS-TER94^RNAi^* flies performed poorly exclusively as a result of disrupted cardiogenesis. VCP is therefore likely required for development of cardiomyocytes and for the maintenance of mature cells and their contractile structures. Preserving structural and functional cellular and subcellular integrity over time is critical to ensure proper performance of post-mitotic tissues characterized by limited regenerative capacity. In skeletal muscle, VCP is essential for myofibrillar development, protein quality control, atrophy, and accelerated degradation [3,7,33,34,35,36,37]. The enzyme appears to perform analogous roles within cardiomyocytes. Interestingly, expression of three MSP-associated disease alleles also disrupted the *Drosophila* heart and influenced its performance, consistent with cardiomyopathy observed in humans with analogous VCP mutations. The restrictive cardiac phenotypes were similar to those recently observed in *Drosophila* with myosin heavy chain and Troponin T mutations [38,39] and did not mirror the effects of excessive RNAi-mediated *TER94* knockdown. The results illustrate that while the disease-associated amino acid substitutions influence the enzyme’s ability to operate normally, as manifested by restricted and poorly functioning cardiac tubes, the mutations did not induce a complete loss of enzymatic function.

VCP participates in diverse biological processes through highly promiscuous interactions with cell-specific cofactors and distinct substrates. Over 50 proteins have been shown to associate either directly or indirectly with VCP [3], although the actual number is likely substantially higher. In skeletal muscle fibers for example, VCP processes have been shown to involve interactions with a host cofactors that include p47, UFD1/Npl4, Ufd2/E4B, and UBXD1 and client proteins such as the myosin chaperone UNC45, caveolin, myosin regulatory light chain, and actin [3,7]. A recent co-immunoprecipitation study revealed associations of VCP with Hsp22, Akt, CryAB, and a limited set of other partners in cardiomyocytes [20]. Multiple lines of evidence suggest alterations in cofactor and substrate interactions, and not a global loss of VCP function, explain MSP molecular pathogenesis [3,4,34,40]. Thus, such perturbations between VCP and cardiomyocyte-specific binding partners are likely triggers for cardiac dysfunction and heart failure, in patients.

Fly models overexpressing *VCP*/*TER94* mutant transgenes via the GAL4-UAS system recapitulate a spectrum of disease phenotypes, including, as evidenced here, cardiac pathology. Thus, as in humans, mutant monomers are seemingly incorporated into and disrupt myocardial roles of the hexameric VCP complex. Impending studies would nevertheless benefit from generating mutant flies via targeted knock-in technology (e.g., CRISPR/Cas9). This would ensure proper timing of gene expression and, potentially, appropriate mutant protein quantities to establish the range of normal and mutant subunit mixtures comprising VCP hexamers to more faithfully recapitulate the diseased state. Since there is remarkable conservation across species of basic VCP functions in non-cardiac tissues [21,26], it is expected that cardiac-specific roles identified in flies are also shared with mice and humans. Therefore, ongoing and future studies, ideally suited for flies, are likely to provide novel insight into VCP’s roles in the heart during health and disease. For example, *Drosophila* is an invaluable tool for rapidly screening for and investigating genetic interactions between proteins of interest. Such experiments, which can be time and cost prohibitive in vertebrates, permit identification and confirmation or rejection of functional interactions between two gene products hypothesized to be in association. They have the potential to help resolve the VCP interactome that dictates the molecule’s functional roles in the heart and to identify possible modifiers of cardiac disease pathogenesis. Therefore, *Drosophila* is an ostensibly powerful model for investigating VCP in cardiomyocytes, resolving its heart-specific interacting partners, and identifying potential modifiers of the response to disease-causing mutations in the enzyme.

## 4. Materials and Methods

### 4.1. Fly Stocks

All flies were raised at 25 °C on a standard cornmeal-yeast-sucrose-agar medium. Cardiac-restricted transgene expression was achieved using the GAL4-UAS bipartite expression system [27]. *UAS-TER94^RNAi^* line (L1) along with its control *w^1118^* strain were obtained from the Vienna *Drosophila* Research Center (Vienna, Austria). A second *UAS-TER94^RNAi^* line (L2), the control *attP2* injection line, and the *TinC-GAL4* cardiac-specific driver line were obtained from the Bloomington *Drosophila* Stock Center (Bloomington, IN, USA). Additional heart-restricted driver lines, *Hand^4.2^-GAL4*, *GMH5-GAL4* and *Hand^4.2^-GS-GAL4* were acquired from Dr. Rolf Bodmer (Sanford Burnham Medical Research Institute, La, Jolla, CA, USA).

Generation of the *UAS-TER94* transgenic lines (*UAS-TER94^WT^*, *UAS-TER94^R152H^*, *UAS-TER94^R188Q^* and *UAS-TER94^A229E^*) is described in Chang *et al.* 2011 [21]. The three mutants model the R155H, R191Q and A232E VCP mutations identified in afflicted human subjects.

For conditional transgene expression, the *Hand^4.2^-GS-GAL4* GeneSwitch driver line was employed. Newly eclosed flies were collected and placed on standard food for two days to permit complete adult cardiac tube development and maturation. From two days of age, flies were placed on food mixed with 100 μg/mL RU486 (dissolved in 100% ethanol) or vehicle (100% ethanol). Flies were transferred onto new food every day to ensure constant exposure to the drug.

### 4.2. Adult *Drosophila* Cardiac Tube Immunohistochemistry

Confocal microscopy of adult female fly hearts was carried out as detailed in Alayari *et al.* 2009 [41]. Beating heart tubes were dissected and exposed under oxygenated adult hemolymph (AH) at 25 °C as described by Vogler *et al.* 2009 [42]. Briefly, the heads, ventral thoraces, and ventral abdominal cuticles were cut and the internal organs and abdominal fat carefully removed, leaving an intact beating heart. Contractions were inhibited by addition of 10 mM EGTA in AH, and hearts were fixed in 4% paraformaldehyde for 30 min, washed three times in PBST, and stained with Alexa594-phalloidin (1:1000) overnight. Cardiac tubes were washed and visualized using a Leica TCS SPE RGBV confocal microscope (Leica Microsystems, Buffalo Grove, IL, USA) at 10× and 40× magnification.

### 4.3. Fluorescence in Situ Hybridization

For quantification of *TER94* transcript levels, beating hearts were dissected and exposed under oxygenated AH at 25 °C [42]. Contractions were inhibited by addition of 10 mM EGTA in AH, and hearts were fixed in 4% paraformaldehyde for 30 min, washed three times in PBST and stored in a 96-well plate in PBS at 4 °C overnight. *In situ* hybridization was performed using the QuantiGene^®^ ViewRNA™ Cell Assay kit from Panomics (Affymetrix Inc., Santa Clara, CA, USA) according to the manufacturer‘s suggested protocol, using sequence specific probes designed to detect *TER94* and *GAPDH* mRNA (catalog numbers VF4-19421 and VF6-18191 respectively). Transcripts were visualized by confocal microscopy with a Leica TCS SPE RGBV confocal microscope (Leica Microsystems, Buffalo Grove, IL, USA) at 40× magnification.

Quantitation of transcripts from confocal micrographs was performed using ImageJ. Distinct fluorescent signals, representing individual *TER94* or *GAPDH* messages, were separated and images converted to 8-bit grayscale. Threshold adjustments were performed for each channel, and were consistently maintained between the different genotypes throughout analysis. Regions of interest, which included only cardiomyocytes, were outlined using the freehand selection option. The number of particles corresponding to individual *TER94* and *GAPDH* transcripts in the same region was determined. The total particle numbers for each set of messages from the different regions of the hearts were normalized to total area. *TER94/GAPDH* ratios were determined and averaged from at least 20 cardiomyocytes for each heart and the ratios from seven or eight hearts averaged for each genotype. Significance was assessed with Student’s *t*-test and significance determined at *p* < 0.05.

### 4.4. Lifespan Analysis

Flies were collected on the day of eclosion and aged at 25 °C in uncrowded vials (25 flies per vial). Fly number was counted and food changed every third day. For each genotype, three independent cohorts from three separate crosses, with 100 flies per set were studied.

### 4.5. Drosophila Cardiac Analysis

Approximately 20–40 cardiac tubes of one-, three-, and five-week-old female *Drosophila* were surgically exposed under AH at 25 °C [42]. Then, 30 second movies were taken at ~120 frames per second using a Hamamatsu Orca Flash 2.8 CMOS camera (Naka-ku, Hamamatsu City, Shizuoka, Japan) on a Leica DM5000B TL miscroscope (Leica Microsystems, Buffalo Grove, IL, USA) with a 10× immersion lens (0.30 NA). Several indices of cardiac performance were measured from the high-speed videos using Semi-automated Optical Heartbeat Analysis (SOHA) (www.sohasofware.com), a free, custom-written motion analysis program [43,44]. Measurements of cardiac diameters at peak diastole and systole were made at two locations along the third abdominal segment of each heart tube, directly from individual movie frames, and averaged together. Significance was assessed using Student’s *t*-tests and one-way ANOVAs with Bonferroni multiple comparison tests.

### 4.6. Drosophila Embryo Immunohistochemistry

*Drosophila* embryos were collected from grape-juice agar plates and aged to the specified stage. Embryos were dechorionated and fixed according to a previously described protocol [45]. The anti-dMef2 antibody (used in 1:750 dilution) was a generous gift from B. Paterson.

## 5. Conclusions

VCP/TER94 is vital for embryonic and adult *Drosophila* heart development and myocardial physiology. As found in other tissues it is abundantly expressed, which likely ensures adequate functional reserve for the enzyme. Thus, mild reductions in VCP/TER94 activity can be tolerated. Excessive loss, however, substantially disrupts myofibrillogenesis and mature cardiomyocyte maintenance. Additionally, expression of MSP disease alleles in the fly heart precipitates cardiomyopathy as observed in patients. The fly permits relatively rapid genome-wide screens via established tools and readily available fly strains to probe cardiac-specific VCP/TER94 interacting partners. Thus, future studies, including such screens, may identify genetic modifiers of VCP-induced cardiomyopathy and therapeutic targets to alleviate the pathophysiological remodeling of the heart that is associated with MSP.

## Supplementary Materials

Supplementary materials can be accessed at http://www.mdpi.com/2308-3425/3/2/19/s1.
