# Supplementary material for: Cardiac-Restricted Expression of VCP/TER94 RNAi or Disease Alleles Perturbs Drosophila Heart Structure and Impairs Function
Source: J Cardiovasc Dev Dis. 2016 May 24;3(2):19. doi: 10.3390/jcdd3020019 (PMC4973812; doi:10.3390/jcdd3020019)
Supplement: Supplementary file 1 [file jcdd-03-00019-s001.pdf]

Supplementary Materials: Cardiac-Restricted Expression of VCP/TER94 RNAi or Disease Alleles Perturbs *Drosophila* Heart Structure and Impairs Function

Meera C. Viswanathan, Anna C. Blice-Baum, Tzu-Kang Sang and Anthony Cammarato

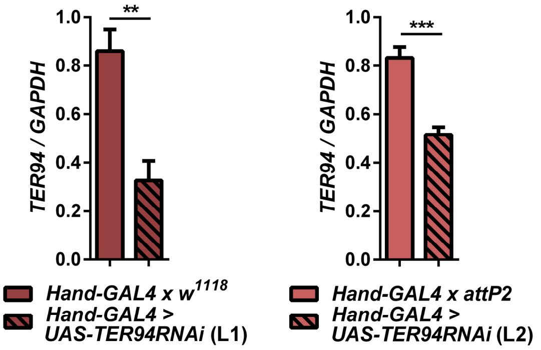

Figure S1. Cardiomyocytes of *Hand-GAL4 > UAS-TER94*^{RNAi} (L1) and *Hand-GAL4 > UAS-TER94*^{RNAi} (L2) *Drosophila* contain reduced *TER94* mRNA levels. ViewRNA FISH was used to quantify *TER94* mRNA levels in fly hearts. As shown in Figure 3, a large reduction in *TER94*/*GAPDH* mRNA signals was determined in *Hand*$^{4.2}$*-GAL4 > UAS-TER94*^{RNAi} (L1) cardimoyocytes compared to *Hand*$^{4.2}$*-GAL4 x w*^{1118} controls (** $p < 0.001$, $n = 7$). *Hand*$^{4.2}$*-GAL4 > UAS-TER94*^{RNAi} (L2) hearts had a similar reduction in *TER94*/*GAPDH* mRNA *vs.* *Hand*$^{4.2}$*-GAL4 x attP2* controls (*** $p < 0.0001$, $n = 8$). Significance was assessed via unpaired *t*-test.

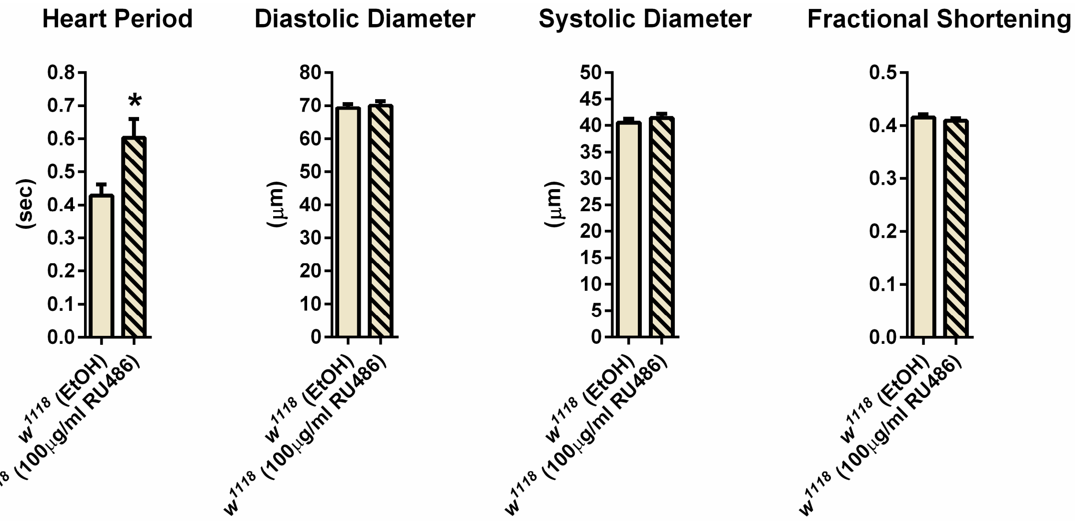

Figure S2. Heart performance of wildtype flies following RU486 treatment post-cardiac development. Semi-automated optical heartbeat analysis was performed on *w*^{1118} flies placed on food supplemented with vehicle (100% ethanol) or 100 μg/mL RU486 (dissolved in 100% ethanol) for three weeks. Flies fed RU486 displayed significantly longer myogenic heart periods. However, relative to flies on vehicle, no differences in systolic diameters, diastolic diameters, or fractional shortening were observed. Thus RU486 does not drastically alter heart performance in flies treated with the compound post-cardiac development. Significance was assessed via unpaired *t*-test, * $p < 0.05$; $n = 15$.
